# Effect of Dietary Supplementation with Lipids of Different Unsaturation Degree on Feed Efficiency and Milk Fatty Acid Profile in Dairy Sheep

**DOI:** 10.3390/ani11082476

**Published:** 2021-08-23

**Authors:** Gonzalo Hervás, Pablo G. Toral, Cristina Fernández-Díez, Antonella Della Badia, Pilar Frutos

**Affiliations:** Instituto de Ganadería de Montaña, CSIC-Universidad de León, Finca Marzanas s/n, 24346 Grulleros, Spain; pablo.toral@csic.es (P.G.T.); cristina.fernandez.diez@csic.es (C.F.-D.); a.dellabadia@csic.es (A.D.B.); p.frutos@csic.es (P.F.)

**Keywords:** ewe, feed conversion ratio, olive oil, palmitic acid, residual feed intake, soybean oil

## Abstract

**Simple Summary:**

The use of fats derived from palm is becoming very common in dairy sheep farms to increase the energy concentration of the diet and therefore the milk production. However, these fats may negatively affect the nutritional quality of milk, whereas feeding unsaturated oils may improve milk fatty acid profile. In this regard, our results in dairy sheep suggested that using palm fat had no evident disadvantage in terms of milk fatty acid composition compared with a diet without supplementation. Nevertheless, it had no positive effects on production or indicators of feed efficiency (for example, milk yield per unit of feed consumed). By contrast, supplementation with oils rich in unsaturated fatty acids (specifically olive oil and soybean oil) improved milk fatty acid profile, with stronger effects with the use of the most unsaturated fat: soybean oil. For example, the latter oil induced the greatest increases in fatty acids with potentially positive effects on human health (e.g., conjugated linoleic acid). In addition, from a practical point of view, the use of soybean oil might also be recommendable to improve the amount of milk produced per unit of feed consumed, compared with the use of palm fat.

**Abstract:**

Lipids of different unsaturation degree were added to dairy ewe diet to test the hypothesis that unsaturated oils would modulate milk fatty acid (FA) profile without impairing or even improving feed efficiency. To this aim, we examined milk FA profile and efficiency metrics (feed conversion ratio (FCR), energy conversion ratio (ECR), residual feed intake (RFI), and residual energy intake (REI)) in 40 lactating ewes fed a diet with no lipid supplementation (Control) or supplemented with 3 fats rich in saturated, monounsaturated and polyunsaturated FA (i.e., purified palmitic acid (PA), olive oil (OO), and soybean oil (SBO)). Compared with PA, addition of OO decreased milk medium-chain saturated FA and improved the concentration of potentially health-promoting FA, such as *cis*-9 18:1, *trans*-11 18:1, *cis*-9 *trans*-11 CLA, and 4:0, with no impact on feed efficiency metrics. Nevertheless, FA analysis and decreases in FCR and ECR suggested that SBO supplementation would be a better nutritional strategy to further improve milk FA profile and feed efficiency in dairy ewes. The paradox of differences observed depending on the metric used to estimate feed efficiency (i.e., the lack of variation in RFI and REI vs. changes in FCR and ECR) does not allow solid conclusions to be drawn in this regard.

## 1. Introduction

In intensive dairy sheep production, feeding systems have moved away from pasture-based to high-concentrate diets, which may affect the nutritional value of milk fat, decreasing the concentration of potentially health-promoting fatty acids (FA), such as *cis*-9 *trans*-11 conjugated linoleic acid (CLA), *trans*-11 18:1, or 18:3n-3 [1,2,3]. In these production systems, diet supplementation with lipids is also widespread to increase the energy density of the ration and therefore production level [4,5,6]. Furthermore, this nutritional strategy has proven to be very useful to improve milk FA profile by modifying the content of bioactive FA [7,8,9].

Supplements rich in palmitic acid (mainly as calcium soaps, palm oil, and fractionated FA) are frequently recommended and used by dairy nutritionists, as they seem to offer the best productive responses [5,10,11]. However, this recommendation is mostly based on knowledge gained from dairy cows and might be related to the susceptibility of this species to milk fat depression (MFD) induced by unsaturated FA (especially when high-concentrate diets are fed) [6,12]. On the contrary, there is evidence that dairy ewes are not prone to this MFD type [3,8,13,14]. Thus, in the ovine, substitution of 16:0-rich fats by oils of higher unsaturation degree (e.g., rapeseed or soybean oils) may provide advantages that go beyond enhancing production level, specifically by modulating milk FA profile [7,15,16].

In the last years, an increasing number of researchers in ruminant nutrition have turned their efforts towards prioritizing an improvement in feed efficiency over production level [17,18,19]. Although there is still very little information on this topic, particularly in dairy ewes [20,21], a recent study has suggested a relationship between feed efficiency and lipid metabolism in the ovine, with certain milk FA being potential biomarkers of this trait (e.g., saturated C4–C14 FA, saturated C4–C14 fatty acids/*cis*-9 18:1 ratio, or C20–22 n-6 polyunsaturated FA) [22]. Thus, because diet composition has a great influence on the efficiency of feed utilization [19,23,24], re-evaluation of the use of lipid supplements aimed at improving milk FA composition is required to examine their effects on metrics and biomarkers of feed efficiency.

On this basis, this study was conducted in dairy ewes to investigate the effect of dietary supplementation with fat sources of different unsaturation degree (i.e., rich in 16:0, in *cis*-9 18:1, or in 18:2n-6) on feed efficiency traits and milk FA composition. Our initial hypothesis was that the use of unsaturated fats to modulate milk FA profile in dairy sheep would not impair or would even improve feed efficiency.

## 2. Materials and Methods

### 2.1. Animals and Management

Forty lactating Assaf ewes were housed in individual tie stalls and fed a total mixed ration (TMR) formulated from dehydrated alfalfa (particle size > 4 cm) and a concentrate (50:50 forage: concentrate ratio). The TMR contained molasses (4% of diet fresh matter) to hinder selection of dietary components. Clean water was always available and fresh diets were offered daily ad libitum after morning milking. Animals were milked twice daily at approximately 08:30 and 18:30 h in a single-side milking parlor with 10 stalls (DeLaval, Madrid, Spain).

After adaptation of the ewes to the TMR (for 1 month) and to the individual tie stalls (for 1 week), feed intake, body weight (BW), and dairy performance were examined over three weeks (pre-experimental period). Then, the 40 sheep were distributed into 4 groups (10 ewes/group) balanced (mean ± SE) for dry matter intake (DMI; 3.70 ± 0.08 kg/day), milk yield (2.59 ± 0.10 kg/day), milk fat and protein concentration (55.0 ± 0.8 and 49.8 ± 0.5 g/kg raw milk, respectively), BW (74.7 ± 1.4 kg), and days in milk (DIM; 61.6 ± 0.7). Groups were randomly allocated to 4 dietary treatments consisting of the basal TMR without lipid supplementation (Control) or supplemented with 2% dry matter (DM) of palm distillate FA (purified commercial product containing 98% of palmitic acid; PA treatment), 2% DM of olive oil (OO treatment) or 2% DM of soybean oil (SBO treatment). These dietary treatments were fed over 4 additional weeks (experimental period). This level of oil supplementation was selected based on their potential modulatory effects on milk FA profile [1,8] and to be practical in terms of cost.

The ingredients and chemical composition of the diets are given in Table 1.

### 2.2. Measurements and Sampling Procedures

#### 2.2.1. Diets

Representative samples of the 4 experimental diets were collected weekly during the pre-experimental and experimental periods (i.e., 7 samples of the basal diet and 4 samples of the supplemented diets). Samples were stored at –30 °C, freeze-dried, and again stored frozen to prevent alterations in fatty acid profile before chemical analysis.

#### 2.2.2. Animal Performance and Feed Efficiency Indicators

To estimate the individual feed efficiency at the pre-experimental and experimental periods, animal performance was monitored over the whole experiment. The BW of each sheep was recorded once weekly.

The DMI was calculated by weighing the amounts of feed offered and refused by each animal. Then, the net energy content of experimental diets (NE_D_) was estimated using the INRA [25] tables of nutritive values of feeds and employed to calculate the net energy intake (NEI = DMI × NE_D_), which is expressed as MJ of net energy/day.

Total milk produced by each ewe at morning and evening milkings was collected and weighed to calculate milk yield. Composite samples of the daily milk produced by each sheep were prepared according to individual yields in morning and evening milkings twice per week (and three times on the last week of each period). One aliquot of that composite milk was preserved with bronopol (D&F Control Systems Inc., San Ramon, CA, USA) and stored at 4 °C until analysis for fat, protein, lactose, and total solid concentrations (within 24–72 h after collection).

On each period, milk yield and milk composition data were used to estimate energy-corrected milk [ECM = kg/d of milk yield × [(0.0071 × g/kg of milk fat) + (0.0043 × g/kg of milk protein) + 0.2224], and net energy requirements for lactation (NE_L_ = 0.686 × ECM, and expressed as MJ of net energy/day), according to INRA [25] equations for sheep. Requirements of protein digestible in the small intestine (PDI) were also estimated according to INRA [25].

The feed conversion ratio (FCR) was calculated as the relationship between mean DMI and ECM on each period, whereas the energy conversion ratio (ECR) was obtained as the relationship between the mean NEI and NE_L_ [26].

Residual feed intake (RFI) on each period was estimated as the residuals of the following regression model [27] using the GLM procedure of the SAS software package (version 9.4; SAS Institute Inc., Cary, NC, USA):

DMI = μ + *a* × ECM + *b* × MBW + *c* × BWC + *d* × DIM + RFI

where DMI represents the mean dry matter intake over the period (kg/day); μ is the intercept; ECM is the energy-corrected milk (kg/day); MBW is the mean metabolic body weight (BW^0.75^; kg); BWC is body weight change over the period (kg); DIM are days in milk; RFI is the residuals; and *a*, *b*, *c*, and *d* are the regression coefficients.

The same procedure was used to estimate the residual energy intake (REI) as the residuals of the following regression model:

NEI = μ + *a* × ECM + *b* × MBW + *c* × BWC + *d* × DIM + REI

where NEI represents the mean net energy intake over the period (MJ/day); μ is the intercept; ECM is the energy-corrected milk (kg/day); MBW is the mean metabolic body weight (BW^0.75^; kg); BWC is body weight change over the period (kg); DIM are days in milk; REI is the residuals; and *a*, *b*, *c*, and *d* are the regression coefficients.

#### 2.2.3. Milk FA Composition

On the last week of each period, aliquots of composite milk from each ewe were collected on 3 consecutive days and stored without preservative at −30 °C until fat extraction for FA composition analysis.

### 2.3. Laboratory Analysis

#### 2.3.1. Experimental Diets

Feed samples were prepared (ISO 6498:2012) and analyzed for DM (ISO 6496:1999), ash (ISO 5984:2002), and crude protein (ISO 5983-2:2009). The concentrations of neutral-detergent fiber (NFD) and acid-detergent fiber (ADF) were sequentially determined using an Ankom^2000^ fiber analyzer (Ankom Technology Methods 13 and 12, respectively; Ankom Technology Corp., Macedon, NY, USA); the former was assayed with sodium sulfite and α-amylase, and both NDF and ADF were expressed with residual ash. Starch content was analyzed by a total starch assay kit obtained from Megazyme (K-TSTA; Megazyme Intl. Ireland Ltd., Wicklow, Ireland).

The fatty acid methyl esters (FAME) of lipid in freeze-dried TMR samples were prepared in a 1-step extraction-transesterification procedure [28], adding 1 mg of *cis*-12 13:1 (10-1301-9, Larodan Fine Chemicals AB, Solna, Sweden) as an internal standard. The methyl esters were separated and quantified using a gas chromatograph (Agilent 7890A GC System, Santa Clara, CA, USA) equipped with a flame ionization detector and a 100 m fused silica capillary column (0.25 mm i.d., 0.2 μm film thickness; CP-SIL 88, Varian Ibérica S.A., Madrid, Spain), and hydrogen as fuel and carrier gas (207 kPa, 2.1 mL/min). Total FAME profile in a 2 μL sample volume at a split ratio of 1:50 was determined using a temperature gradient program [28]: following sample injection, column temperature was maintained at 70 °C for 4 min, increased at a rate of 8 °C/min to 110 °C, raised to 170 °C at a rate of 5 °C/min, held at 170 °C for 10 min, increased at 4 °C/min to a final temperature of 240 °C that was maintained for 14.5 min. Peaks were identified based on retention time comparisons with commercially available standards (GLC463, Nu-Chek Prep, Elysian, MN, USA; 18919-1AMP Supelco, Sigma-Aldrich, Madrid, Spain).

#### 2.3.2. Milk Composition

Milk samples were analyzed for fat, protein, lactose, and total solid concentration by infrared spectrophotometry (ISO 9622:1999) using a MilkoScan FT6000 (Foss, Hillerød, Denmark).

Lipids in 1 mL of milk were extracted and converted to FAME by base-catalyzed transesterification [28]. Total FAME profile was determined using the same chromatograph and temperature gradient program applied for the analysis of feed, but isomers of 18:1 were further resolved in a separate analysis under isothermal conditions at 170 °C [26]. All peaks were identified based on retention time comparisons with commercially available standards (GLC463, U-37-M, U-43-M, U-45-M and U-64-M, from Nu-Chek Prep; 18919-1AMP Supelco, L6031, L8404 and O5632, from Sigma-Aldrich; and 11-1600-8, 20-2024-1, 20-2210-9, 20-2305-1-4, 21-1211-7, 21-1413-7, 21-1614-7, 21-1615-7 and BR mixtures 2 and 3, from Larodan Fine Chemicals AB), with reference samples for which the FA composition was determined based on gas chromatography analysis of FAME and GC–MS analysis of corresponding 4,4-dimethyloxazoline derivatives [29,30], and with chromatograms reported in the literature [28].

### 2.4. Statical Analysis

Statistical analysis was performed using the MIXED procedure of SAS software package (version 9.4).

Data were analyzed by one-way analysis of covariance with a model that included the fixed effect of the 4 experimental treatments (Control, PA, OO and SBO) and measurements on the pre-experimental period as a covariate, as follows:

*y_ijk_* = μ + α*_i_* + *d_j(i)_* + (*b* + φ*_j_*) *x_ij_* + e*_ijk_*

where *y_ijk_* is the dependent variable measured at time *k* (experimental period) on the *j*th animal assigned to the *i*th diet, μ the overall mean effect, α*_i_* the *i*th fixed diet effect, *d_j(i)_* the random effect of the *j*th animal within the *i*th diet, *b* the common regression coefficient of initial value of *x_ij_*, φ*_j_* the slope deviation of the *i*th diet from common slope *b*, *x_ij_* the initial record measure (pre-experimental period) of the *j*th animal on the *i*th diet, and e*_ijk_* the random error associated with the *j*th animal assigned to the *i*th diet at time *k*.

Means were separated through the pairwise differences (pdiff) option of the least squares means (lsmeans) statement of the MIXED procedure and adjusted for multiple comparisons using Bonferroni’s method. Differences were declared significant at *p* < 0.05 and considered a trend toward significance at 0.05 ≤ *p* < 0.10. Least squares means are reported.

## 3. Results

### 3.1. Animal Performance and Feed Efficiency Indicators

As shown in Table 2, diet supplementation with lipids affected FCR, with a 12% decrease in SBO treatment compared with the Control and PA (*p* = 0.012). Similarly, ECR tended to be 11% lower in SBO than in PA treatment (*p* = 0.052). On the contrary, residual traits (RFI and REI) were not significantly modified by the inclusion of lipids in the TMR (*p* > 0.10).

Feed intake tended to be affected by diet (*p* = 0.066), but no differences or trends toward difference were observed in pairwise comparisons after adjustment using Bonferroni’s method. Body weight and yields of milk, ECM, protein, lactose and total solids remained unaffected by treatment (*p* > 0.10). However, compared with the Control, milk fat yield was 12% greater in OO (*p* = 0.039), with an increase in the molar production of <C16 and >C16 FA (*p* < 0.01). Supplementation with SBO also improved milk > C16 FA yield compared with the Control (*p* < 0.001), whereas the production of C16 FA was greater in PA than in other treatments (*p* < 0.001). In addition, milk fat content was 10 and 13% higher in OO and SBO, respectively, compared with the Control (*p* = 0.001), but no significant effects were observed in the concentration of milk protein, lactose, and total solids.

Protein balance was positive in the four experimental treatments: ewes consumed on average 127 ± 3% of their estimated PDI requirements.

### 3.2. Milk Short- and Medium-Chain FA

Table 3 reports the content of milk short- and medium-chain FA, which were differently affected by lipid supplementation. Specifically, ewes on PA treatment showed the greatest proportions of 16:0 and *cis*-9 16:1 in milk (*p* < 0.001), but 12:0, 14:0, and *cis*-7 14:1 concentrations were lower than in the Control (*p* < 0.001). Reductions in these medium-chain FAs were greater in OO and SBO treatments, which showed the lowest content of most FAs with 10 to 16 carbon atoms, such as 10:0, *cis*-9 12:1, and 16:0 (*p* < 0.05), except for the increase in *trans*-9 16:1 in SBO relative to other diets (*p* < 0.001) and the lack of variation in *cis*-9 10:1 and *trans*-5 to -8 16:1 (*p* > 0.10). Compared with the Control, the milk concentration of 4:0 was increased in OO and SBO (*p* < 0.001), and that of 6:0 in SBO (*p* = 0.001). On average, lipid supplements caused an 11% decrease in the sum of saturated C4-C14 FA (i.e., those mostly derived from mammary *de novo* synthesis) relative to the Control (*p* < 0.001).

### 3.3. Milk C18 FA

Dietary treatments showed clearly divergent effects of milk C18 FA (Table 4), and most FA within this group were more abundant in OO, and specially in SBO, compared with control and PA (*p* < 0.05). For example, 18:0, *trans*-9 and *trans*-10 18:1, or *trans*-10 *trans*-14 18:2 were similarly increased by the two unsaturated lipid supplements (*p* < 0.001), but SBO caused the greatest increment in the concentrations of *trans*-11 18:1, other 18:1 isomers with Δ12 to Δ16 double bonds, non-conjugated 18:2 isomers, and *cis*-9 *trans*-11, *trans*-9 *cis*-11 and *trans*-10 *cis*-12 CLA (*p* < 0.01). However, the highest proportions of *cis*-9 18:1 and of the minor 10-oxo-18:0 and *trans*-4 to *trans*-8 18:1 were found in OO treatment (*p* < 0.001). This latter oil negatively affected the percentage of milk *cis*-9 *cis*-12 18:2 and *cis*-9 *cis*-12 *cis*-15 18:3 (*p* < 0.001), whereas SBO improved *cis*-9 *cis*-12 18:2 content (*p* < 0.001). On the other hand, *cis*-11 18:1, *trans*-11 *cis*-13 CLA and *trans*-9 *trans*-12 *trans*-15 18:3 remained unaffected by dietary treatment (*p* > 0.10).

Finally, the ratio between saturated C4-C14 FA and *cis*-9 18:1 was 34% lower in OO and SBO than in Control and PA treatments (*p* < 0.001).

### 3.4. Other Milk FA

Very long-chain FA are reported in Table 5. Compared with the Control, PA only affected (i.e., decreased) 24:0 concentration, which was also reduced in OO treatment (*p* = 0.004). Milk 20:2n-6, 22:5n-6, and the sum of C20–22 n-6 polyunsaturated FA were greater in PA than OO (*p* = 0.034). On the contrary, this latter treatment and SBO resulted in the greatest milk concentration of *cis*-11 and *trans*-11 20:1, and 20:4n-3 and *cis*-13 22:1 were increased in SBO treatment (*p* < 0.01).

The sums of milk odd- and branched-chain FA were negatively affected by the inclusion of unsaturated oils (*p* < 0.001; Table 6). Regarding individual FA within these two groups, the content of 11:0, *iso* 13:0, 15:0, *anteiso* 15:0, *iso* 15:0, or 21:0 decreased in OO and SBO relative to the Control (*p* < 0.05), whereas *anteiso* 13:0 and *anteiso* 17:0 were only reduced in OO (*p* < 0.05), and 4,8,12-trimethyl-13:0 increased in SBO relative to other treatments (*p* = 0.002). On the other hand, PA caused no significant variation in milk odd- and branched-chain FA compared with the Control, except for a decrease in 23:0 (*p* < 0.001).

## 4. Discussion

In this study, lipids of different unsaturation degree were added to dairy ewe diet to test the hypothesis that unsaturated oils would modulate milk FA profile without impairing or even improving feed efficiency. To this aim, we examined the responses to 3 vegetable fats rich in saturated, monounsaturated, and polyunsaturated FA (i.e., 16:0, *cis*-9 18:1, and 18:2n-6, respectively). Although their main effects on milk FA profile have been previously described [8,9,15], we report a comprehensive FA composition because available profiles in the literature are often poorly detailed, especially in terms of minor C18, odd-, branched-, and very long-chain FA. Although their biological effects are largely unknown [31,32,33], a lack of detail in presentation of results may limit the future advancement of knowledge or the potential application of FA as noninvasive biomarkers [22,34,35].

The use of 16:0-rich supplements, widely spread in cattle production, is increasingly common in dairy sheep farms under intensive conditions [5,15]. These fats are very effective at improving the energy density of the ration without negatively affecting nutrient digestibility [11,36], but their effects on milk FA profile might offer some drawbacks [7,16,36]. In our study, we observed an increment in the milk concentration of 16:0 with PA, consistent with expectations [7,15]. Although increasing 16:0 consumption might pose a greater risk of cardiovascular disease for human consumers [37,38], such effect might be counteracted by the inversely proportional impact of PA on milk 14:0 and 12:0, which have also been reported to be atherogenic [39]. In addition, PA caused virtually no alteration in the concentration of other bioactive FA in milk, either potentially negative (e.g., *trans*-9 and *trans*-10 18:1) or positive (e.g., *cis*-9 *trans*-11 CLA and *trans*-11 18:1), in agreement with its potential inertness in the rumen and lower toxicity for microbiota than unsaturated FA [36,40,41]. Thus, our results would support that using palmitic-rich products in dairy sheep feeding has no evident disadvantage in terms of milk fat quality. Nevertheless, it does not appear to offer any advantage in terms of efficiency of feed utilization, according to the lack of variation in the studied metrics compared with the control, both in ratio traits (i.e., FCR and ECR) and in residual traits (i.e., RFI and REI).

Similarly, OO treatment had neither positive nor negative consequences on feed efficiency indicators, despite improvements in milk fat concentration and yield. We used olive oil as a model of fat rich in monounsaturated FA (specifically, *cis*-9 18:1), due to its easy and ready availability in most intensive dairy sheep production areas (in particular, in the Mediterranean basin) and its close FA profile to that of other lipid supplements widely studied in ruminant nutrition (e.g., rapeseed oil) [42,43,44]. Regarding the impact of OO on milk fat composition, it is worth highlighting some desirable effects, such as the decrease in medium-chain saturated FA and the increase in some potentially health-promoting compounds, specifically 4:0, *trans*-11 18:1, *cis*-9 *trans*-11 CLA, and *cis*-9 18:1 [1,39]. The large variation in the latter would derive not only from dietary *cis*-9 18:1 supply, but also from its extensive saturation in the rumen [45,46], enhancing the availability of 18:0 for mammary Δ^9^-desaturation [47]. Ruminal *cis*-9 18:1 metabolism also involves isomerization and hydration/oxidation processes [45,46], which would partly explain the increments in milk *trans* 18:1 and 10-oxo-18:0, respectively. In addition, changes in 18:1 isomers may also derive from a greater biohydrogenation extent of 18:2n-6 and 18:3n-3, as suggested by the drop in their milk concentration. This effect on biohydrogenation extent has been consistently described in studies on ruminal metabolism when unsaturated FA supplements are provided [46,48]. On the contrary, certain effects of OO on milk FA profile were less desirable, in particular the increase in *trans*-9 and *trans*-10 18:1 or the decrease in branched-chain FA, which would be explained by direct isomerization of *cis*-9 18:1 in the rumen or inhibition of microbial de novo FA synthesis, respectively [35,40,45].

Among the treatments studied, SBO showed the best potential to modulate milk FA profile. Compared with OO, it induced even greater improvements in *cis*-9 *trans*-11 CLA and *trans*-11 18:1 concentrations, with similar variations in medium-chain saturates and other potentially bioactive FA (e.g., 4:0 and *trans*-10 18:1). Moreover, SBO improved 18:2n-6 and had no negative effect on 18:3n-3. Although this may increase the n-6/n-3 FA ratio in milk, the implications of this index for human health are under debate, and focusing attention on improving the consumption of both types of polyunsaturated FA is increasingly encouraged [49,50]. On the other hand, SBO was the only treatment that raised milk *trans*-10 *cis*-12 CLA content, but its final proportion was actually marginal (0.006% of total FA) and, therefore, no MFD was induced. A recent meta-analysis has indeed shown that much higher *trans*-10 *cis*-12 CLA concentrations may be reached (~0.031% of total FA) without risk of MFD in sheep fed high-concentrate diets and plant oils, given their ability to compensate the inhibition of de novo FA synthesis by enhanced preformed FA yield [51].

In addition, the reduction in FCR with SBO suggests an improvement in the efficiency of feed utilization compared with the Control and PA treatments. In this regard, the comparison between SBO and PA is particularly interesting, as they are isoenergetic diets. This would explain the consistency in the SBO vs. PA comparison when the ECR was employed, an indicator that is estimated using the net energy intake, whereas the FCR is based on DM intake [18,26]. Thus, ECR seems more convenient in our study because it avoids the bias associated to the different energy density of our experimental diets [26]. However, when residual traits (RFI and REI) were examined, no variation was detected and responses to supplemented treatments did not follow a similar pattern to that observed with ratio traits.

Residual traits are currently more recommended and used as indicators of feed efficiency in genetic selection [19,22,27]; their interest deriving from their potential relationship with basic metabolic processes [52,53]. In Australia, steers from low-RFI selection lines have been shown to consume less feed for the same level of growth performance and, thus, improve the profitability of farms [24]. Nevertheless, from a productive point of view and with the perspective of a direct application in the dairy sector, decreased FCR and ECR would also entail economic advantages for farmers, thus the potentially positive implications of our findings. Furthermore, animal performance data suggest that the lower FCR and ECR in SBO would partly be explained by increased FA yield, which supports a key role of lipid metabolism in underlying feed efficiency mechanisms [22,54]. Further, note that our results did not seem to be explained by mobilization of body reserves, since all treatments showed improved body weight during the trial.

Finally, regarding a validation of previously suggested biomarkers of feed efficiency in dairy ewes (e.g., saturated C4-C14 FA, saturated C4-C14 fatty acids/*cis*-9 18:1 ratio or C20–22 n-6 polyunsaturated FA in milk) [22], no solid conclusions can be drawn. The reason is none other than the divergent effects of experimental diets on the milk concentration of these biomarkers. Thus, for example, OO and SBO treatments caused both increases and decreases in individual even-chain saturated C4-C14 FA, which may bias their total amount in milk. In addition, the improvement in *cis*-9 18:1 concentration in the same treatments would be explained by the additional dietary supply of this monounsaturated FA and the greater mammary availability of its precursor, 18:0, rather than a greater mobilization of adipose tissue (rich in *cis*-9 18:1) in animals under negative energy balance [55,56]. Therefore, treatment differences in saturated C4-C14 fatty acids/*cis*-9 18:1 ratio cannot actually be related to potential variations in feed efficiency when animals fed different lipid supplements are compared [22]. In any event, the results of this experimental trial would not undermine the application of suggested biomarkers in dairy sheep farms, where all lactating ewes would be offered the same diet. Otherwise, discriminating animals by feed efficiency level should be conducted independently within each dietary condition.

## 5. Conclusions

Overall, our results support the initial hypothesis that unsaturated lipid supplements modulate milk FA profile in dairy sheep without impairing or even improving feed efficiency. Compared with a saturated fat rich in 16:0 (palm distillate FA), addition of a source of monounsaturated FA (olive oil) decreases medium-chain saturated FA in milk and improves the concentration of potentially health promoting FA, such as *cis*-9 18:1, *trans*-11 18:1, *cis*-9 *trans*-11 CLA, and 4:0, with no impact on feed efficiency indicators. Nevertheless, results of FA analysis and decreases in FCR and ECR suggest that using soybean oil supplementation would be a more convenient nutritional strategy to achieve further improvements in milk FA profile and also in feed efficiency in dairy ewes. However, the paradox of differences observed depending on the metric used to estimate feed efficiency (i.e., the lack of variation in residual traits—RFI and REI—vs. changes in ratio traits—FCR and ECR) does not allow solid conclusions to be drawn in this regard.

## Figures and Tables

**Table 1:** Formulation and chemical composition of the experimental diets.

	Diet			
	Control	PA	OO	SBO
Ingredients, g/kg of fresh matter				
Dehydrated alfalfa, particle size > 4 cm	500	491	491	491
Whole corn grain	140	138	138	138
Whole barley grain	100	98	98	98
Soybean meal, solvent 440 g crude protein/kg	150	147	147	147
Sugar beet pulp, pellets	50	49	49	49
Molasses, liquid	40	39	39	39
Vitamin-mineral supplement ^1^	20	20	20	20
Oil supplement ^2^	0	18	18	18
Composition, g/kg diet dry matter (except for dry matter itself; g/kg of fresh matter)				
Dry matter	900	906	902	901
Organic matter	908	908	906	909
Crude protein	182	176	173	171
Neutral detergent fiber	302	293	301	303
Acid detergent fiber	215	213	215	216
Starch	130	144	130	137
Total fatty acids	22.95	41.44	41.42	41.44
14:0	0.13	0.28	0.13	0.14
16:0	5.10	23.77	7.50	7.07
*cis*-9 16:1	0.04	0.04	0.26	0.06
18:0	0.85	0.85	1.38	1.42
*cis*-9 18:1	3.39	3.33	16.50	7.69
*cis*-11 18:1	0.20	0.20	0.74	0.51
*cis*-9 *cis*-12 18:2	9.42	9.23	10.85	19.26
*cis*-9 *cis*-12 *cis*-15 18:3	2.99	2.93	3.06	4.27
20:0	0.19	0.19	0.27	0.25
22:0	0.18	0.18	0.21	0.27
24:0	0.24	0.23	0.25	0.26

^1^*MACROFAC Rumiantes* (UP911755130; DSM Nutritional Products S.A., Madrid, Spain). Declared as containing: Ca (285 g/kg), Na (7.5 g/kg), Fe (3 g/kg), Mn (3 g/kg), Zn (2 g/kg), Mg (1 g/kg), P (910 mg/kg), Mo (100 mg/kg), Co (67 mg/kg), I (50 mg/kg), S (40 mg/kg), Se (7 mg/kg), vitamin A (200,000 IU/kg), vitamin D3 (40,000 IU/kg), vitamin E (667 mg/kg), ethoxyquin (12 mg/kg), and propyl gallate (2 mg/kg). ^2^ PA: palm distillate fatty acids (SOLAFAM 440, AFAMSA S.A., Mos, Pontevedra, Spain); OO: pure and refined olive oil (Carrefour SA, Madrid, Spain); SBO: soybean oil (OLI-BEEF; INATEGA S.L. Corbillos de la Sobarriba, León, Spain).

**Table 2:** Animal performance in dairy ewes fed a total mixed ration without lipid supplementation (Control) or supplemented with 2% dry matter (DM) of palm distillate fatty acids (PA), olive oil (OO), and soybean oil (SBO).

	Diet					
	Control	PA	OO	SBO	SED ^1^	*p*-Value
Feed conversion ratio (FCR)	1.76 ^a^	1.73 ^a^	1.67 ^ab^	1.54 ^b^	0.07	0.012
Energy conversion ratio (ECR)	2.39	2.48	2.39	2.21	0.09	0.052
Residual feed intake (RFI)	0.014	−0.100	0.045	−0.114	0.094	0.233
Residual energy intake (REI)	−0.036	1.362	0.317	0.528	0.819	0.353
DM intake, kg/d	3.32	3.26	3.33	3.06	0.11	0.066
Body weight, kg	75.4	75.5	75.0	76.5	0.6	0.122
Body weight change, kg	7.2	5.7	7.3	3.7	1.4	0.044 ^2^
Yield, kg/d						
Milk	2.40	2.42	2.48	2.35	0.10	0.590
Energy corrected milk (ECM)	2.01	2.08	2.13	2.11	0.09	0.611
Fat	0.132 ^b^	0.138 ^ab^	0.150 ^a^	0.144 ^ab^	0.006	0.039
Protein	0.118	0.115	0.121	0.114	0.004	0.415
Lactose	0.122	0.121	0.126	0.118	0.005	0.528
Total solids	0.395	0.396	0.422	0.399	0.016	0.296
Fatty acid yield, mmol/d						
Total fatty acids	541 ^c^	579 ^bc^	660 ^a^	633 ^ab^	28	<0.001
<C16	283 ^b^	292 ^b^	337 ^a^	320 ^ab^	15	0.004
C16	148 ^b^	172 ^a^	145 ^b^	135 ^b^	8	<0.001
>C16	113 ^b^	115 ^b^	177 ^a^	177 ^a^	11	<0.001
Milk composition, g/kg raw milk						
Fat	54.8 ^c^	56.8 ^bc^	60.3 ^ab^	61.7 ^a^	1.7	0.001
Protein	48.6	47.6	49.1	48.5	0.9	0.388
Lactose	50.6	49.7	50.7	50.3	0.7	0.475
Total solids	163.8	163.3	169.7	170.2	2.7	0.016 ^2^

^a–c^ Within a row, different superscripts indicate differences (*p* < 0.05) due to the effect of diet. ^1^ SED = standard error of the difference. ^2^ In the pairwise analysis, no significant differences were found after adjustment for multiple comparisons using Bonferroni’s method.

**Table 3:** Milk short- and medium-chain fatty acids (g/100 g of total fatty acids) in dairy ewes fed a total mixed ration without lipid supplementation (Control) or supplemented with 2% dry matter (DM) of palm distillate fatty acids (PA), olive oil (OO), and soybean oil (SBO).

	Diet				SED ^1^	*p*-Value
	Control	PA	OO	SBO		
4:0	3.24 ^b^	3.39 ^ab^	3.44 ^a^	3.53 ^a^	0.07	<0.001
6:0	2.86 ^b^	2.85 ^b^	3.04 ^ab^	3.10 ^a^	0.07	0.001
8:0	3.00	2.86	3.13	3.10	0.11	0.074
10:0	10.90 ^a^	9.93 ^ab^	9.65 ^b^	9.43 ^b^	0.38	0.003
*cis*-9 10:1	0.305	0.291	0.288	0.284	0.016	0.576
12:0	6.95 ^a^	6.04 ^b^	5.09 ^c^	5.05 ^c^	0.31	<0.001
*cis*-9 12:1	0.122 ^a^	0.111 ^a^	0.083 ^b^	0.081 ^b^	0.008	<0.001
*trans*-9 12:1	0.056 ^a^	0.052 ^a^	0.042 ^b^	0.041 ^b^	0.003	<0.001
14:0	13.21 ^a^	11.69 ^b^	10.74 ^c^	10.61 ^c^	0.34	<0.001
*cis*-7 14:1	0.022 ^a^	0.019 ^b^	0.017 ^bc^	0.015 ^c^	0.001	<0.001
*cis*-9 14:1	0.195 ^a^	0.177 ^ab^	0.152 ^b^	0.151 ^b^	0.011	<0.001
*cis*-12 14:1	0.110 ^a^	0.102 ^a^	0.078 ^b^	0.073 ^b^	0.007	<0.001
16:0	28.94 ^b^	32.84 ^a^	24.98 ^c^	24.25 ^c^	0.75	<0.001
*trans*-5 16:1	0.028	0.028	0.028	0.023	0.002	0.027 ^2^
*trans*-6 + 7 + 8 16:1	0.106	0.093	0.130	0.126	0.019	0.152
*trans*-9 16:1	0.054 ^b^	0.060 ^b^	0.086 ^b^	0.146 ^a^	0.013	<0.001
*cis*-9 16:1	0.758 ^b^	0.849 ^a^	0.666 ^c^	0.649 ^c^	0.032	<0.001
*cis*-11 16:1	0.016 ^a^	0.015 ^a^	0.012 ^b^	0.012 ^b^	0.001	<0.001
*cis*-13 16:1	0.013 ^a^	0.012 ^a^	0.009 ^b^	0.010 ^b^	0.001	<0.001
∑ saturated C4-C14 fatty acids	40.14 ^a^	36.81 ^b^	35.07 ^b^	34.81 ^b^	0.99	<0.001

^a–c^ Within a row, different superscripts indicate differences (*p* < 0.05) due to the effect of diet. ^1^ SED = standard error of the difference. ^2^ In the pairwise analysis, no significant differences were found after adjustment for multiple comparisons using Bonferroni’s method.

**Table 4:** Milk C18 fatty acids (g/100 g of total fatty acids) in dairy ewes fed a total mixed ration without lipid supplementation (Control) or supplemented with 2% dry matter (DM) of palm distillate fatty acids (PA), olive oil (OO), and soybean oil (SBO).

	Diet				SED ^1^	*p*-value
	Control	PA	OO	SBO		
18:0	6.10 ^b^	5.57 ^b^	9.51 ^a^	8.41 ^a^	0.43	<0.001
10-oxo-18:0	0.012 ^bc^	0.006 ^c^	0.024 ^a^	0.018 ^ab^	0.003	<0.001
13-oxo-18:0	0.007 ^a^	0.003 ^b^	0.004 ^ab^	0.005 ^ab^	0.001	0.017
*cis*-9 18:1 ^2^	10.43 ^c^	10.62 ^c^	15.56 ^a^	13.66 ^b^	0.65	<0.001
*cis*-11 18:1	0.329	0.349	0.388	0.356	0.023	0.106
*cis*-12 18:1	0.234 ^b^	0.221 ^b^	0.254 ^b^	0.670 ^a^	0.033	<0.001
*cis*-13 18:1	0.052 ^c^	0.047 ^c^	0.066 ^b^	0.085 ^a^	0.004	<0.001
*cis*-15 18:1	0.085 ^bc^	0.080 ^c^	0.101 ^b^	0.153 ^a^	0.006	<0.001
*cis*-16 18:1	0.038 ^bc^	0.034 ^c^	0.046 ^b^	0.071 ^a^	0.003	<0.001
*trans*-4 18:1	0.015 ^c^	0.013 ^c^	0.061 ^a^	0.033 ^b^	0.004	<0.001
*trans*-5 18:1	0.011 ^c^	0.009 ^c^	0.046 ^a^	0.027 ^b^	0.003	<0.001
*trans*-6 + 7 + 8 18:1	0.158 ^c^	0.154 ^c^	0.574 ^a^	0.403 ^b^	0.028	<0.001
*trans*-9 18:1	0.142 ^b^	0.122 ^b^	0.391 ^a^	0.337 ^a^	0.023	<0.001
*trans*-10 18:1	0.232 ^b^	0.212 ^b^	0.490 ^a^	0.548 ^a^	0.027	<0.001
*trans*-11 18:1	0.597 ^c^	0.639 ^bc^	1.119 ^b^	1.888 ^a^	0.177	<0.001
*trans*-12 18:1	0.258 ^c^	0.241 ^c^	0.541 ^b^	0.647 ^a^	0.029	<0.001
*trans*-15 18:1	0.188 ^c^	0.175 ^c^	0.292 ^b^	0.396 ^a^	0.021	<0.001
*trans*-16 + *cis*-14 18:1	0.292 ^c^	0.259 ^c^	0.385 ^b^	0.525 ^a^	0.020	<0.001
*cis*-9 *cis*-12 18:2	2.33 ^b^	2.26 ^b^	1.81 ^c^	2.71 ^a^	0.09	<0.001
*cis*-9 *trans*-12 18:2	0.033 ^c^	0.030 ^c^	0.044 ^b^	0.064 ^a^	0.004	<0.001
*cis*-9 *trans*-13 18:2 ^3^	0.198 ^c^	0.185 ^c^	0.257 ^b^	0.372 ^a^	0.017	<0.001
*cis*-9 *trans*-14 18:2	0.100 ^c^	0.096 ^c^	0.128 ^b^	0.175 ^a^	0.007	<0.001
*trans*-9 *cis*-12 18:2	0.025 ^bc^	0.024 ^c^	0.031 ^b^	0.047 ^a^	0.002	<0.001
*trans*-11 *cis*-15 + *trans*-10 *cis*-15 18:2	0.063 ^b^	0.057 ^b^	0.060 ^b^	0.116 ^a^	0.009	<0.001
*trans*-12 *cis*-15 18:2	0.014 ^b^	0.013 ^b^	0.015 ^b^	0.023 ^a^	0.002	<0.001
*trans*-10 *trans*-14 18:2	0.012 ^b^	0.010 ^b^	0.018 ^a^	0.018 ^a^	0.001	<0.001
*trans*-11 *trans*-15 18:2	0.012 ^b^	0.011 ^b^	0.016 ^b^	0.028 ^a^	0.002	<0.001
*cis*-9 *trans*-11 CLA ^4^	0.325 ^c^	0.334 ^c^	0.554 ^b^	0.880 ^a^	0.077	<0.001
*trans*-9 *cis*-11 CLA	0.013 ^b^	0.012 ^b^	0.017 ^ab^	0.021 ^a^	0.002	0.001
*trans*-10 *cis*-12 CLA	0.003 ^b^	0.003 ^b^	0.003 ^b^	0.006 ^a^	0.001	0.002
*trans*-11 *cis*-13 CLA ^5^	0.011	0.010	0.010	0.013	0.002	0.433
*trans*-11 *trans*-13 CLA	0.053 ^ab^	0.058 ^a^	0.034 ^c^	0.043 ^bc^	0.005	<0.001
∑ other *trans*, *trans* CLA ^6^	0.011 ^b^	0.010 ^b^	0.017 ^a^	0.017 ^a^	0.002	<0.001
*cis*-9 *cis*-12 *cis*-15 18:3	0.667 ^a^	0.643 ^a^	0.482 ^b^	0.635 ^a^	0.029	<0.001
*cis*-9 *trans*-11 *trans*-15 18:3	0.006 ^b^	0.006 ^b^	0.006 ^b^	0.012 ^a^	0.001	<0.001
*cis*-9 *trans*-12 *cis*-15 18:3	0.012 ^b^	0.013 ^ab^	0.016 ^a^	0.014 ^ab^	0.001	0.035
*trans*-9 *cis*-12 *cis*-15 18:3 ^7^	0.007 ^b^	0.006 ^b^	0.011 ^a^	0.013 ^a^	0.001	<0.001
*trans*-9 *trans*-12 *trans*-15 18:3	0.002	0.002	0.005	0.002	0.002	0.258
saturated C4-C14 fatty acids/*cis*-9 18:1	3.88 ^a^	3.58 ^a^	2.31 ^b^	2.62 ^b^	0.23	<0.001

^a–c^ Within a row, different superscripts indicate differences (*p* < 0.05) due to the effect of diet. ^1^ SED = standard error of the difference. ^2^ Coelutes with *trans*-13 + 14 18:1. ^3^ Coelutes with *cis*-10 *trans*-14, *trans*-10 *trans*-13, and *trans*-11 *trans*-14 18:2. ^4^ Contains *trans*-7 *cis*-9 and *trans*-8 *cis*-10 CLA as minor isomers. ^5^ Coelutes with an unidentified component. ^6^ Sum of *trans*-8 *trans*-10, *trans*-9 *trans*-11, and *trans*-10 *trans*-12 CLA. ^7^ Coelutes with *cis*-5 20:1.

**Table 5:** Milk very long-chain fatty acids (g/100 g of total fatty acids) in dairy ewes fed a total mixed ration without lipid supplementation (Control) or supplemented with 2% dry matter (DM) of palm distillate fatty acids (PA), olive oil (OO), and soybean oil (SBO).

	Diet				SED ^1^	*p*-Value
	Control	PA	OO	SBO		
20:0 ^2^	0.274	0.268	0.271	0.281	0.011	0.694
*cis*-8 + 9 20:1	0.011	0.010	0.010	0.011	0.001	0.069
*cis*-11 20:1	0.037 ^b^	0.036 ^b^	0.050 ^a^	0.046 ^a^	0.002	<0.001
*trans*-11 20:1	0.003 ^b^	0.003 ^b^	0.008 ^a^	0.006 ^a^	0.001	<0.001
20:2n-6	0.017 ^ab^	0.018 ^a^	0.015 ^b^	0.017 ^ab^	0.001	0.009
20:3n-6	0.024	0.025	0.022	0.026	0.002	0.233
20:3n-3	0.008	0.007	0.008	0.006	0.001	0.449
20:4n-6	0.152 ^a^	0.149 ^a^	0.120 ^b^	0.146 ^ab^	0.010	0.009
20:4n-3	0.001 ^b^	0.001 ^b^	0.001 ^b^	0.003 ^a^	0.000	<0.001
20:5n-3	0.049 ^ab^	0.058 ^a^	0.042 ^b^	0.044 ^b^	0.004	<0.001
22:0	0.090 ^ab^	0.078 ^bc^	0.075 ^c^	0.096 ^a^	0.005	<0.001
*cis*-13 22:1	0.003 ^b^	0.004 ^b^	0.004 ^b^	0.009 ^a^	0.001	<0.001
22:4n-6	0.021	0.021	0.017	0.022	0.002	0.058
22:5n-6	0.010 ^ab^	0.014 ^a^	0.008 ^b^	0.012 ^a^	0.001	0.001
22:5n-3	0.088	0.098	0.083	0.088	0.008	0.312
22:6n-3	0.024	0.025	0.025	0.028	0.003	0.593
24:0	0.037 ^a^	0.031 ^b^	0.029 ^b^	0.032 ^ab^	0.002	0.004
*cis*-15 24:1	0.010	0.009	0.008	0.007	0.001	0.192
∑C20–22 n-6 polyunsaturated fatty acids	0.225 ^ab^	0.229 ^a^	0.189 ^b^	0.219 ^ab^	0.014	0.034
∑C20–22 n-3 polyunsaturated fatty acids	0.082	0.090	0.075	0.081	0.006	0.098

^a–c^ Within a row, different superscripts indicate differences (*p* < 0.05) due to the effect of diet. ^1^ SED = standard error of the difference. ^2^ Coelutes with 18:3n-6.

**Table 6:** Milk odd- and branched-chain fatty acids (g/100 g of total fatty acids) in dairy ewes fed a total mixed ration without lipid supplementation (Control) or supplemented with 2% dry matter (DM) of palm distillate fatty acids (PA), olive oil (OO), and soybean oil (SBO).

	Diet				SED ^1^	*p*-Value
	Control	PA	OO	SBO		
5:0	0.020	0.021	0.019	0.019	0.001	0.527
7:0	0.045	0.044	0.040	0.045	0.003	0.341
9:0	0.077	0.073	0.065	0.066	0.006	0.084
11:0	0.124 ^a^	0.107 ^ab^	0.085 ^b^	0.087 ^b^	0.010	0.001
*anteiso* 13:0	0.010 ^ab^	0.010 ^a^	0.008 ^b^	0.008 ^ab^	0.001	0.019
*iso* 13:0	0.024 ^a^	0.019 ^ab^	0.016 ^b^	0.015 ^b^	0.003	0.012
*iso* 14:0	0.099	0.093	0.081	0.078	0.008	0.031^2^
15:0	0.938 ^a^	0.858 ^a^	0.711 ^b^	0.742 ^b^	0.033	<0.001
*anteiso* 15:0	0.392 ^a^	0.375 ^ab^	0.318 ^c^	0.329 ^bc^	0.021	0.003
*iso* 15:0 ^3^	0.219 ^a^	0.199 ^ab^	0.183 ^b^	0.175 ^b^	0.013	0.005
*cis*-9 15:1	0.011	0.010	0.009	0.010	0.001	0.303
*trans*-6 + 7 15:1	0.020	0.021	0.017	0.020	0.002	0.080
*iso* 16:0	0.221 ^a^	0.203 ^ab^	0.173 ^b^	0.200 ^ab^	0.015	0.023
4,8,12-trimethyl-13:0	0.056 ^b^	0.057 ^b^	0.056 ^b^	0.066 ^a^	0.003	0.002
17:0	0.516	0.511	0.456	0.466	0.022	0.016 ^2^
*anteiso* 17:0	0.420 ^a^	0.406 ^a^	0.353 ^b^	0.391 ^ab^	0.017	0.003
*iso* 17:0 ^4^	0.591	0.564	0.553	0.567	0.023	0.418
*cis*-9 17:1	0.173	0.176	0.147	0.148	0.012	0.030 ^2^
*iso* 18:0	0.048	0.049	0.037	0.042	0.006	0.149
19:0 ^5^	0.088 ^a^	0.080 ^ab^	0.075 ^b^	0.085 ^ab^	0.004	0.016
21:0 ^6^	0.071 ^a^	0.064 ^ab^	0.057 ^b^	0.060 ^b^	0.004	0.003
23:0	0.064 ^a^	0.052 ^b^	0.045 ^b^	0.047 ^b^	0.004	<0.001
∑odd-chain fatty acids	2.15 ^a^	2.02 ^a^	1.72 ^b^	1.80 ^b^	0.06	<0.001
∑branched-chain fatty acids	2.10 ^a^	2.00 ^ab^	1.78 ^c^	1.89 ^bc^	0.06	<0.001

^a-c^ Within a row, different superscripts indicate differences (*p* < 0.05) due to the effect of diet. ^1^ SED = standard error of the difference. ^2^ In the pairwise analysis, no significant differences were found after adjustment for multiple comparisons using Bonferroni’s method. ^3^ Contains *trans*-9 14:1 as a minor isomer. ^4^ Coelutes with *cis*-7 16:1. ^5^ Coelutes with *trans*-9 *trans*-12 18:2. ^6^ Coelutes with *trans*-12 *trans*-14 CLA.
